# A Single-Dataset-Based Pre-Processing Joint Domain Localized Algorithm for Clutter-Suppression in Shipborne High-Frequency Surface-Wave Radar

**DOI:** 10.3390/s20133773

**Published:** 2020-07-05

**Authors:** Liang Guo, Xin Zhang, Di Yao, Qiang Yang, Yang Bai, Weibo Deng

**Affiliations:** 1 School of Electronics and Information Engineering, Harbin Institute of Technology, Harbin 150001, China; 16B905009@stu.hit.edu.cn (L.G.); zhangxinhit@hit.edu.cn (X.Z.); yq@hit.edu.cn (Q.Y.); hit_baiyang@163.com (Y.B.); 2 Key Laboratory of Marine Environmental Monitoring and Information Processing, Ministry of Industry and Information Technology, Harbin 150001, China; yaodi1988@126.com; 3 School of Information Science and Engineering, Harbin Institute of Technology at Weihai, Weihai 264209, China

**Keywords:** signal processing, space time adaptive processing, clutter-suppression

## Abstract

Due to the motion of the platform, the spectrum of first-order sea clutter will widen and mask low-velocity targets such as ships in shipborne high-frequency surface-wave radar (HFSWR). Limited by the quantity of qualified training samples, the performance of the generally used clutter-suppression method, space–time adaptive processing (STAP) degrades in shipborne HFSWR. To deal with this problem, an innovative training sample acquisition method is proposed, in the area of joint domain localized (JDL) reduced-rank STAP. In this clutter-suppression method, based on a single range of cell data, the unscented transformation is introduced as a preprocessing step to obtain adequate homogeneous secondary data and roughly estimated clutter covariance matrix (CCM). The accurate CCM is calculated by integrating the approximate CCM of different range of cells. Compared with existing clutter-suppression algorithms for shipborne HFSWR, the proposed approach has a better signal-to-clutter-plus-noise ratio (SCNR) improvement tested by real data.

## 1. Introduction

In shipborne high-frequency surface-wave radar (HFSWR), the main detection background clutter for targets of low-velocity is the spreading first-order sea clutter caused by the platform motion. Under this circumstance, it is necessary to suppress the spread clutter for target detecting.

The effect of platform motion on the spectra of the received signals has been noted and some analyses have been carried out [1]. Xie, Yuan and Liu gave the spreading model of the first-order sea clutter and the theoretical analyses match well with the experimental results. The eigenspectral structure of the first-order sea clutter was also demonstrated [2], which shows the possibility of using space–time adaptive processing (STAP) to suppress sea clutter in shipborne HFSWR. Sun et al. developed a formula that can accurately estimate the rank of ocean clutter covariance matrix [3]. The first-order and second-order high-frequency radar cross section (RCS) models of the ocean surface were derived for HFSWR on a floating platform with sway motion by Walsh, J., Huang, W. and Gill, E. [4,5,6,7]. The first-order and second-order ocean-surface cross sections for shipborne HFSWR with uniform linear motion and sway motion were derived by Xie, Sun and Ji [8,9,10]; (J) E. Khoury et al. presented a model of received signal from a floating HFSWR and assessed the effect of antenna motion on the first-order Bragg lines [11]. Over the years, the effects of forward motion, sway motion, surge motion, rotation motion and other motions caused by ocean dynamics on both monostatic and bistatic shipborne HFSWR have been studied [12,13,14,15,16,17]. These analyses provide guidance for target detection in shipborne HFSWR. A target detection technique with the aid of synthetic aperture theory was put forward by Gao and Zong [18] but it has strict requirements on the motion state. The orthogonal weighting (OW) algorithm and the oblique projection (OP) algorithm, which separate targets and clutter through projecting them to different subspaces, were presented [19,20]. These two methods suppress the sea clutter in space domain, and the space–time coupling properties of the first-order sea clutter are not taken into consideration. Generally, STAP will get better results when suppressing space-time coupled clutter. The optimum STAP algorithm needs to know the exact clutter covariance matrix (CCM) and target space–time steering vector [21]. However, the CCM is generally unknown. The most common method is to estimate the CCM using the training samples adjacent to the cell under test (CUT) which have independent and identically distributed (IID) clutter. According to the Reed–Mallett–Brennan (RMB) criterion, the amount of training samples should also be sufficient to achieve an average signal to noise ratio (SNR) loss of less than three decibels [22]. For shipborne HFSWR, a possible architecture of the STAP processor is described and simulated in [23]. A practical STAP method named the joint domain localized (JDL) algorithm was introduced into shipborne HFSWR in [24]. Simulations show good Doppler results can be obtained. However, it assumes that the clutter has a uniform distribution in range dimension. Its performance is restricted by the existence of estimation error of the CCM. A modified JDL algorithm was proposed in [25], which utilities the correlation coefficients as weights to calculate CCM. The training samples which are similar to the CUT counts more and the proportion of unrelated training samples are weakened. An improved orthogonal weighting (IOW) algorithm [26] and an improved oblique projection (IOP) algorithm [27] were proposed, despite significant progress in the direction of arrival estimation, these JDL-like methods use samples in Doppler domain to estimate the CCM and suffer from the same drawbacks as the conventional JDL algorithm.

Due to limited qualified training samples, the performance of STAP algorithms encounters a major challenge, that is, the estimated CCM is inaccurate. In order to degenerate this performance degradation, recent developments mainly focused on two approaches, one is looking for efficient training samples selecting methods to pick out samples most similar to the CUT. These selected samples are used to estimate the CCM, such as the generalized inner product (GIP) method [28], the power-selected training (PST) method [29] and waveform similarity method [30,31,32,33,34]. These methods acquire better results while they may require sufficient initial training samples. The other way is using sparse reconstruction technique to constructing the CCM as shown in [35,36,37,38,39,40,41,42,43]. However, the performance of the CCM reconstruction method in real data seems to be poor, although the simulated situations are perfect.

For shipborne HFSWR, there are not enough training samples for several reasons. First, constrained by the size, the detecting range is small. In addition, HFSWR mainly focuses on Doppler frequency resolution and its range resolution is always low, which will lead to a small quantity of training samples. Even worse, the first-order sea clutter echo spreads and occupies a wide range of Doppler frequencies. Different from onshore HFSWR, clutter energy is not concentrated on a narrow Bragg frequency. As a result, the statistical characteristics of the clutter vary significantly in range dimension, which means that the training samples in range dimension are not IID. Thus, not only the quantity of the training samples is small but also the quality is bad. To deal with this problem, a single-dataset-based preprocessing JDL algorithm is proposed in this study. which uses an innovative training sample acquisition method. Like the conventional JDL method, the received array data are transformed into the angle-Doppler domain and the size of the local processing region (LPR) is determined according to statistical characteristics. It is worth mentioning that this part is not the focus of this paper, therefore, this paper directly uses the conclusions drawn in our previous conference paper [25]. After that the unscented transformation is deployed to obtain more available training samples based on the data of a single range cell, and the CCM of this CUT is roughly approximated. A more accurate CCM of the CUT is estimated by integrating the CCM of different range of cells. The suboptimal weight vector is formulated on this CCM. Finally, the spread clutter is suppressed using the STAP weight vector in the LPR.

The structure of this paper is organized as follows. In Section 2, the basic theory of the conventional JDL algorithm is given as necessary background. The shipborne HFSWR system is briefly introduced in Section 3.1. Based on this, the space-time distribution of the spread first-order sea clutter and the homogeneity in range dimension is analyzed in Section 3.2 and Section 3.3. The results show that the first-order sea clutter is nonhomogeneous in range dimension, and the application of JDL in shipborne HFSWR should be adjusted. In Section 4, the proposed single-dataset-based preprocessing JDL algorithm is presented which gives a better method for using JDL in shipborne HFSWR. Section 5 provides the experimental results and performance comparison between the proposed method with existing algorithms. Discussion is in Section 6. Finally, the conclusion is addressed in Section 7.

## 2. Principle of Joint Domain Localized Processing Algorithm

JDL processing is one of the reduced-dimension STAP methods which is used to suppress clutter in homogeneous environments. To facilitate subsequent analysis, the signal model in conventional JDL is illustrated. This model can be found in [24,30,32,44,45,46].

**Figure 1:**
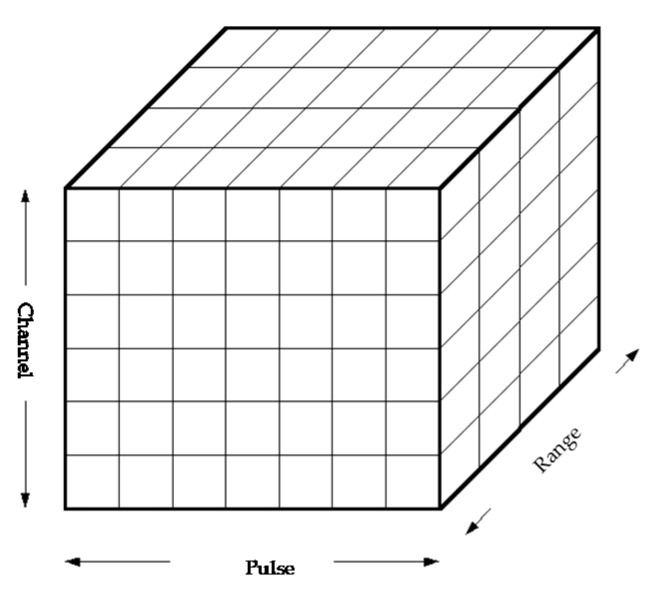
Data model.

Figure 1 illustrates the received data model in HFSWR. The received array data are preprocessed to obtain range samples. For a uniform linear array (ULA) of *N* isotropic monopole antennas in the entire coherent processing interval (CPI) of *M* pulses, the data of the *i*th range cell is

(1) $$X_{i}=\left[\begin{array}{cccc} x_{1,1} & x_{1,2} & \cdots & x_{1,M}\\ x_{2,1} & x_{2,2} & \cdots & x_{2,M}\\ \vdots & \vdots & \ddots & \vdots \\ x_{N,1} & x_{N,2} & \cdots & x_{N,M} \end{array}\right]$$

where $x_{nm}$ represents the sampled data in the *n*th channel and *m*th pulse. $x_{i}=vec(X_{i})$ denotes the vectorization of the matrix $X_{i}$ formed by stacking the columns of $X_{i}$ into a single column vector.

According to the echo component, this vector $x_{i}$ consists of three parts,

(2) x=s+c+n

Among them, $s=\sum_{k=1}\sigma_{k}v_{k}(\theta_{k},f_{k})$, $c=\sum_{j=1}\sigma_{j}v_{j}(\theta_{j},f_{j})$ represents the targets and clutter, respectively. n denotes the thermal noise. σ is the amplitude which represents the intensity of the echo energy, θ, f denotes the azimuth and Doppler frequency, respectively. v is the steering vector of the space–time point (θ,f). It can be decomposed into Kronecker products of the spatial steering vector a(θ) and the temporal steering vector b(f), as follows:

(3) v(θ,f)=b(f)⊗a(θ)

(4) $$a(\theta )={\left[1\;e^{j2\pi \cdot f_{s}}\;e^{j2\pi \cdot 2f_{s}}\;\ldots \;e^{j2\pi \cdot (N-1)f_{s}}\right]}^{T}$$

(5) $$b(f)={\left[1\;e^{j2\pi \cdot f_{t}}\;e^{j2\pi \cdot 2f_{t}}\;\ldots \;e^{j2\pi \cdot (M-1)f_{t}}\right]}^{T}$$

In which ⊗ denotes the Kronecker product of two vectors, $f_{s}=(d/\lambda )\sin\theta$ is the normalized spatial frequency, λ is the operating wavelength.$f_{t}=f/f_{R}$ is the normalized temporal frequency, $f_{R}$ is the pulse repetition frequency (PRF). ${\left[\cdot \right]}^{T}$ denotes transposition.

In conventional JDL processing, a transformation matrix T is used to transform the vectorized receiving data x (NM×1) and the space-time steering vector v(θ,f) (NM×1) into a LPR ($\eta_{a}\times \eta_{d}$) in the angle-Doppler domain. The central point of the LPR is (θ,f). Let $\tilde{x}$, $\tilde{v}$ denotes the transformed data and steering vector, this transformation is written as

(6) $${\tilde{x}}_{l}=T^{H}\cdot x_{l},\;\tilde{v}(\theta ,f)=T^{H}\cdot v(\theta ,f).$$

Take a 3×3 ($\eta_{a}=3,\eta_{d}=3$) rectangle LRP centered at $\left(\theta_{i},f_{i}\right)$ as an example. The transformation matrix is

(7) $$T_{\left(\theta_{i},f_{i}\right)}=\left[b\left(f_{i-1}\right)b\left(f_{i}\right)b\left(f_{i+1}\right)\right]⊗\left[a\left(\theta_{i-1}\right)a(\theta )a\left(\theta_{i+1}\right)\right].$$

where the subscript ${\left[\cdot \right]}_{i-m}$ indicates the point to the left or down of the central point ${\left[\cdot \right]}_{i}$ with the distance equals to m, ${\left[\cdot \right]}_{i+m}$ indicates the point to the right or up of the central point ${\left[\cdot \right]}_{i}$ with the distance m.

The transformed data vector ${\tilde{x}}_{l}$, after reshaping to a matrix, takes the following form:

(8) $${\tilde{X}}_{l}=\left[\begin{array}{ccc} \begin{array}{c} {\tilde{x}}_{\left(\theta_{i+1},f_{i-1}\right)}\\ {\tilde{x}}_{\left(\theta_{i},f_{i-1}\right)}\\ {\tilde{x}}_{\left(\theta_{i-1},f_{i-1}\right)} \end{array} & \begin{array}{c} {\tilde{x}}_{\left(\theta_{i+1},f_{i}\right)}\\ {\tilde{x}}_{\left(\theta_{i},f_{i}\right)}\\ {\tilde{x}}_{\left(\theta_{i-1},f_{i}\right)} \end{array} & \begin{array}{c} {\tilde{x}}_{\left(\theta_{i+1},f_{i+1}\right)}\\ {\tilde{x}}_{\left(\theta_{i},f_{i+1}\right)}\\ {\tilde{x}}_{\left(\theta_{i-1},f_{i+1}\right)} \end{array} \end{array}\right].$$

Then the CCM is estimated as

(9) $${\hat{\tilde{R}}}_{l}=\frac{1}{K}\sum_{p=0,p\neq l}^{K-1}{\tilde{x}}_{p}{\tilde{x}}_{p}^{H},K\geq 2\eta_{a}\eta_{d}$$

The adaptive weights can be obtained from the following equation

(10) $${\tilde{w}}_{l}={\hat{\tilde{R}}}_{l}^{-1}\tilde{v}$$

The output after clutter-suppression is

(11) $$y={\tilde{w}}_{l}^{H}{\tilde{x}}_{l}$$

In this study, decision statistics are calculated using the modified sample matrix inversion (MSMI) statistics,

(12) $$\eta_{MSMI}=\frac{{\left|w^{H}x\right|}^{2}}{w^{H}v}\begin{array}{c} >\\ < \end{array}\;\eta_{0}\;\begin{array}{c} H1\\ H0 \end{array}$$

where $\eta_{0}$ denotes the detection threshold in a constant false-alarm rate (CFAR) detector. $H_{0}$ and $H_{1}$ represent the target-absent and present hypotheses, respectively. If the decision statistic $\eta_{MSMI}=\frac{{\left|w^{H}x\right|}^{2}}{w^{H}v}$ is greater than the threshold $\eta_{0}$, it means that a target is present. Otherwise, the target is absent.

## 3. Heterogeneity of the First-Order Sea Clutter

The premise to use JDL for clutter-suppression is that the clutter is homogeneous. In the study [24], no analysis of the first-order sea clutter is found, which recognized this premise by default. In order to obtain better performance, characteristics of the first-order sea clutter is analyzed in this section. The experimental results based on real data show that the first-order sea clutter is not uniform in range dimension. Therefore, when applied to shipborne HFSWR, the traditional JDL approach needs to be adjusted or modified. The structure of this section is as follows. The real shipborne HFSWR system is briefly introduced in Section 3.1. The space-time distribution of the first-order sea clutter is given in Section 3.2. The homogeneity of the first-order sea clutter in range dimension is analyzed in Section 3.3 by using correlation coefficients.

### 3.1. Shipborne HFSWR System

An experiment was conducted and real data were recorded from the shipborne HFSWR system in the Yellow Sea of China in September 1998. As described in [18,19,24], the system parameters are shown in Table 1.

**Figure 2:**
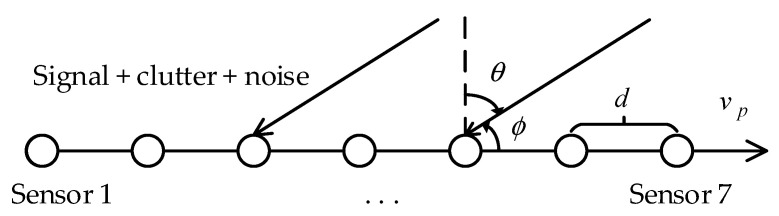
Receiving array [24].

The receiving array of the shipborne HFSWR system consists of 7 receiving antennas which form a ULA. The entire system is mounted on a barge which is tugged by a tugboat. Like the side-looking airborne radar, the radar points perpendicular to the direction of the flight. Figure 2 depicts the sketch map of the receiving array.

### 3.2. Space-Time Distribution of the First-Order Sea Clutter

**Figure 3:**
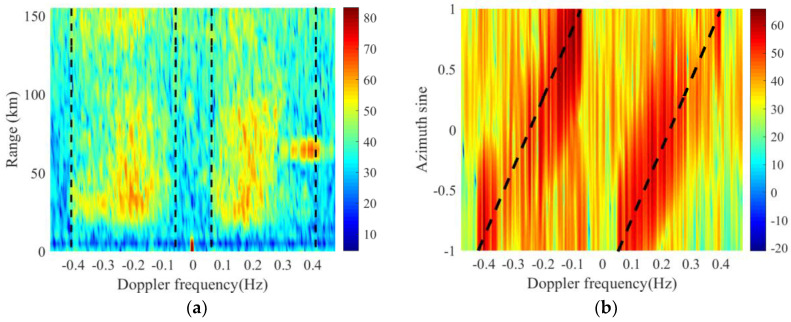
Example of real data. (**a**) Range-Doppler map of the shipborne high-frequency surface-wave radar (HFSWR), -- -- denotes for the theoretical range of spreading first-order sea clutter; (**b**) angle-Doppler map of the shipborne HFSWR, -- -- denotes for the theoretical value of first-order sea clutter.

In shipborne HFSWR, the principal component of the detecting background for low velocity targets is the spreading first-order sea clutter. Xie, Yuan and Liu [2] analyzed the space–time distribution of the first-order sea clutter based on the real data and derived the space–time distribution of the first-order sea clutter spectrum as:

(13) $$f_{d}=\pm f_{B}+f_{dp}\cos\phi$$

where $f_{B}\approx 0.102\sqrt{f_{0}}$ denotes the first-order Bragg frequency, $f_{0}$ represents the carrier frequency of the radar. $f_{dp}=2v_{p}/\lambda$ is the Doppler frequency of the platform, θ is the incident angle of the clutter patch, ϕ is the complementary angle of θ and ϕ=π/2 −θ,ϕ∈[0,π]. Here we choose real data to show this phenomenon. After Doppler processing and beamforming, the channel–pulse–range data cube is transformed into azimuth–Doppler-range data cube. The range-Doppler map and angle-Doppler map of data file 1128 are selected and illustrated in Figure 3. As we can see, the first-order sea clutter spreads to a range of Doppler frequencies and there is a linear relationship between the azimuth sine and the Doppler frequency of the clutter.

### 3.3. Homogeneity Analysis of the First-Order Sea Clutter in Range Dimension

The premise to obtain CCM using other range cells is the clutter is homogeneous in range dimension. However, this assumption fails in shipborne HFSWR.

**Figure 4:**
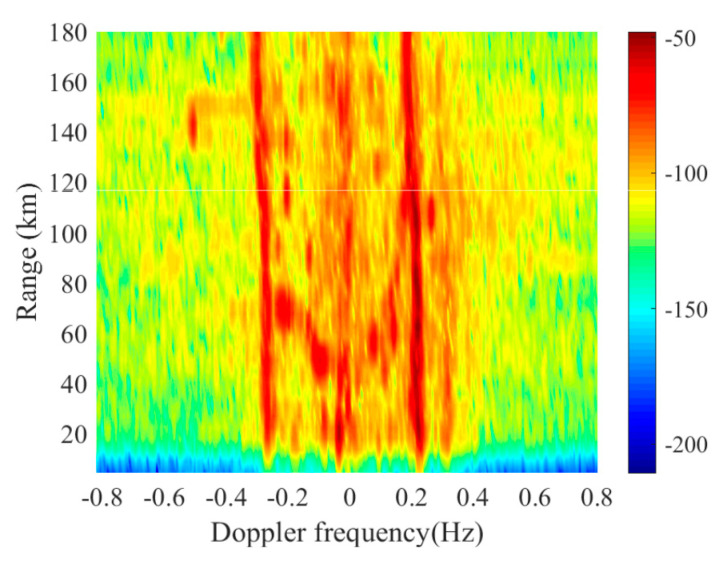
Example of the range-Doppler map of the onshore HFSWR.

When the platform stays still, like onshore HFSWR, the first-order sea clutter concentrates to the Bragg frequency and can be seen as an approximately straight line in the range-Doppler map. Considering that the strength of the first-order sea clutter is so high that the clutter takes the dominant position and their statistic characteristics can be seen as homogeneous. For an intuitive standpoint, Figure 4 illustrates this distribution with real data. These data come from the onshore HFSWR system in Weihai, China on 12 May 2012. Compared with Figure 3a, it is obvious that the spectrum of the first-order sea clutter is not concentrated at the Bragg frequency in shipborne HFSWR. The strength of the first-order sea clutter also fluctuates in range dimension for shipborne HFSWR. Thus, the first-order sea clutter from different range of cells do not share same features. Adding the CCM of different ranges directly will not get an accurate estimation.

**Figure 5:**
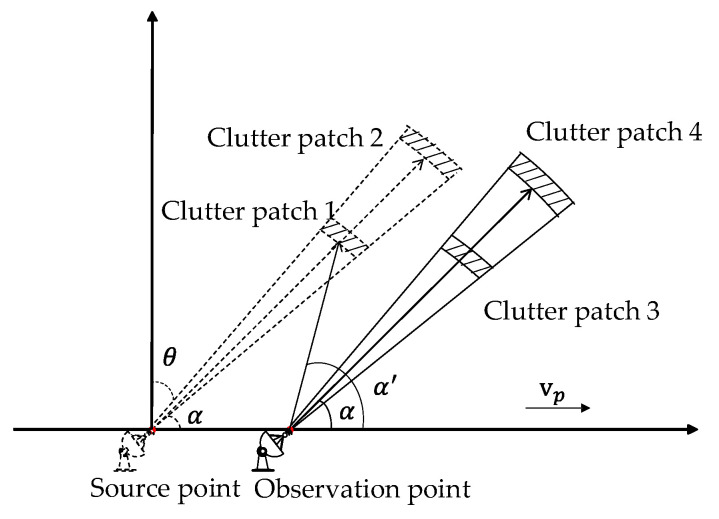
Shipborne HFSWR scatter geometry.

The reason for this may be as follows. The sea clutter from a range cell can be modeled as the superposition of a large number of independent clutter patches that are evenly distributed in azimuth [3]. When the platform moves, the source point and the observation point are not the same in a coherent integration time (CIT) and the clutter patches differ. As shown in Figure 5, we define the direction of arrival (DOA) as α, the echoes come from scatter points in different ranges like clutter patch 1 and clutter patch 2 at the source point when the integrated period starts. When the platform moves to the observation point at the end of the CIT, the DOA of the clutter patch 1 changes to $\alpha^{\prime }$. If we keep the DOA α the same, like the situation in the range-Doppler map in Figure 3a, the scatter points change. Thus, it may cause the fluctuation of the statistic characteristic in range dimension.

To evaluate the homogeneity of the spreading first-order sea clutter in range dimension, the correlation coefficients between the CUT and training samples from other range cells are calculated. Here, we choose the correlation analysis method proposed in [32]. The received array data are transformed into the angle-Doppler domain and the LPR is determined according to the statistic characteristics. The correlation characteristic in angel dimension and Doppler dimension was analyzed in our previous work [25]. The size of the LPR is determined as three angle bins and three Doppler bins, where the interval is $5^{{}^{\circ}}$ and 0.0037 Hz. The correlation analysis method based on azimuth–Doppler joint eigenvectors is as follows:

1. Vectorize the data ${\tilde{X}}_{\mathrm{LPR}k}$ by ${\tilde{x}}_{\mathrm{LPR}k}=vec({\tilde{X}}_{\mathrm{LPR}k})$ in the *k*th LPR. The covariance matrix ${\tilde{R}}_{{\tilde{X}}_{\mathrm{LPR}k}}$ can be calculated as ${\tilde{R}}_{{\tilde{X}}_{\mathrm{LPR}k}}={\tilde{x}}_{\mathrm{LPR}k}{\tilde{x}}_{\mathrm{LPR}k}^{H}$;
2. Eigen-decompose ${\tilde{R}}_{{\tilde{X}}_{\mathrm{LPR}k}}$ and obtain 9 eigenvalues $\lambda_{1},\lambda_{2},\ldots ,\lambda_{9}$ and 9 eigenvectors $\xi_{1},\xi_{2},\ldots ,\xi_{9}$, then pick out the eigenvector corresponding to the largest eigenvalue as $\varsigma_{k}$;
3. Compute the correlation coefficients by the following equation $\rho_{i}=\varsigma_{k}^{H}\varsigma_{i},\;i=1,2,\ldots ,K$.

**Figure 6:**
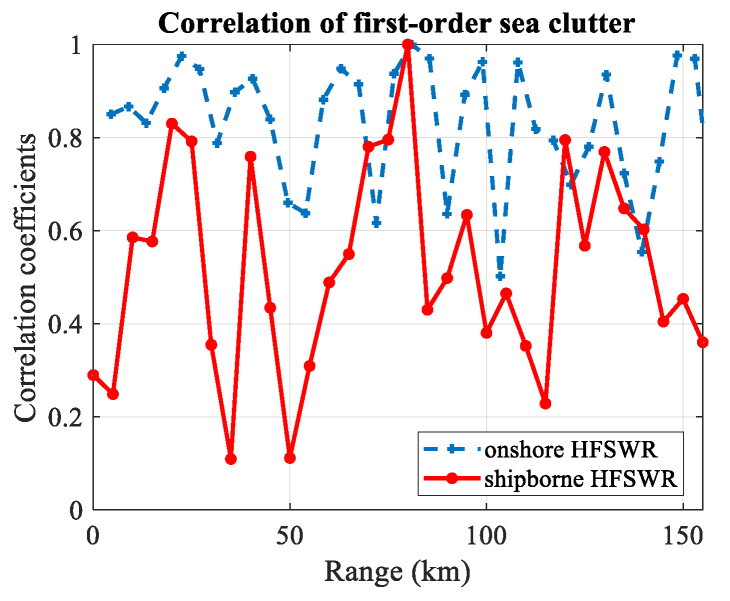
Correlation of first-order sea clutter in range dimension.

The correlation coefficients between the CUT and other range bins are shown in Figure 6. The solid line marked with a circle sign represents for the shipborne HFSWR and the dashed line marked with a plus sign shows the correlation in onshore HFSWR. The reference range bin is at 80 km. We can see that the correlation coefficients in onshore HFSWR are almost above 0.6, while the correlation coefficients in shipborne HFSWR are much lower and irregularly distributed. This result clearly proves that the first-order sea clutter is not homogeneous in range dimension.

In conclusion, the first-order sea clutter in shipborne HFSWR is broaden and has a specific space-time distribution. It is nonhomogeneous in range dimension. Clutter-suppression methods need to consider these features.

## 4. Modified JDL Algorithm with Training Samples Acquisition Method

STAP is an efficient method to deal with space–time coupled clutter. The fully STAP utilizes the CCM of the CUT to calculate the optimal weight. Generally, the CCM is unknown and needs to be estimated from training samples. For shipborne HFSWR, the number of receiving channel *N* is often two to dozens and the number of pulses *M* is often thousands to several tens of thousands. For example, the number of pulses is 1024 to achieve a long CIT for higher Doppler resolution and the number of channels is 7 in the shipborne HFSWR system used in this paper. In this scenario, at least 14,336 training samples are needed for fully STAP, which is unavailable.

Therefore, dimensional-reduction STAP methods are introduced. The JDL algorithm is one of the suboptimum STAP methods which decrease the amount of training samples through transforming the data to the angle-Doppler domain. It is widely used in finite training samples situation. Like the other suboptimum STAP algorithms, the training samples which are used to estimate the CCM should be target free and contain clutter with the same statistical properties as the CUT. The performance of the JDL algorithm degrades drastically when the estimation of the CCM is inaccurate. The minimum number of samples required to achieve a robust performance is twice the degree of freedom of the CUT, according to the RMB criterion [22]. In this paper, at least 18 IID samples are required for a 3×3 LPR. This requirement cannot always be secured because of the clutter heterogeneities.

To deal with this problem, a single-dataset-based preprocessing JDL algorithm is proposed here. The unscented transformation is introduced to preprocess the primary data to obtain adequate homogeneous secondary data and approximate the CCM initially. Then the rough approximation of CCM from different range bins is integrated together to obtain a more accurate result which will also increase the rank of the CCM since it can be seen as a way of smoothing. The proposed data acquisition method can generate 2 *N* + 1 secondary training samples based on an *N* dimensional primary data. It is worth mentioning that the generated secondary samples cannot replace the primary samples. However, it can improve the performance to some extent.

### 4.1. Training Sample Acquisition Method

In nonhomogeneous scenario like shipborne HFSWR, the lack of qualified training samples is the major factor which influences the accuracy of CCM estimation. Using the adjacent range cells directly to estimate the CCM will not get a good result. If we can obtain some homogeneous secondary data from the CUT data through a transformation and approximate the covariance of the CUT by using the produced secondary data, the accuracy of the estimation will be improved. This procedure can be seen as a special transformation. The input data are the CUT and the expected output is more available training data. Thus, the unscented transformation is suitable here.

The unscented transformation is a method to estimate the statistic parameters of a nonlinear transform [47]. It approximates a probability distribution with a set of sample points which are called sigma points. These points parameterize the mean and covariance of the distribution. When nonlinearly transformed, these transformed points completely capture the mean and covariance of the new density. The procedure of the unscented transform is as follows. Consider a single nonlinear transformation y=h(x) with a n×1 dimension stochastic vector x, the mean and the covariance of x is $m_{x}$ and $R_{x}$, respectively. Select *N* sigma points $\sigma_{x}^{i},i=0,1,2,\ldots ,N-1$. Transform the sigma points through the known nonlinear function $\sigma_{y}^{i}=h(\sigma_{x}^{i}),i=0,1,2,\ldots ,N-1$. the true mean and covariance of the output y can be approximated by the weighted sample mean and sample covariance of the $\sigma_{y}^{i}$. Denote the weight associated with the $i^{th}$ sigma point as $W_{m}^{\left(i\right)}$, the estimate of the output mean is

(14) $${\hat{m}}_{y}=\sum_{i=0}^{N-1}W_{m}^{\left(i\right)}\sigma_{y}^{i}.$$

Denoted the weight with the covariance as $W_{c}^{\left(i\right)}$ and the estimate of the output covariance is

(15) $${\hat{R}}_{y}=\frac{1}{N}\sum_{i=0}^{N-1}W_{c}^{\left(i\right)}(\sigma_{y}^{i}-{\hat{m}}_{y}){\left(\sigma_{y}^{i}-{\hat{m}}_{y}\right)}^{T}.$$

For general unscented transformation, 2*n* + 1 sigma points are used, which follows the formula

(16) $$\begin{array}{l} \sigma_{x}^{\left(0\right)}=m_{x}\\ \sigma_{x}^{\left(i\right)}=m_{x}+{\tilde{R}}_{x}^{\left(i\right)},i=1\ldots ,2n \end{array}$$

${\tilde{R}}_{x}^{\left(i\right)}$ satisfy the following equations

(17) $$\begin{array}{l} {\tilde{R}}_{x}^{\left(i\right)}={\left(\sqrt{(n+\lambda )R_{x}}\right)}_{i}^{T},i=1\ldots ,n\\ {\tilde{R}}_{x}^{\left(n+i\right)}={\left(\sqrt{(n+\lambda )R_{x}}\right)}_{i}^{T},i=1\ldots ,n \end{array}$$

where λ is the scaling parameter, $\lambda =\alpha^{2}(n+\kappa )-n$. The parameters α∈(0,1] and κ≥0 influence how far the sigma points are away from the mean. ${\left(\sqrt{(n+\lambda )R_{x}}\right)}_{i}$ represents the $i^{th}$ row of $\sqrt{(n+\lambda )R_{x}}$. $\sqrt{(n+\lambda )R_{x}}$ is the matrix square root of $(n+\lambda )R_{x}$.

The weights $W_{m}^{\left(0\right)}$ and $W_{c}^{\left(0\right)}$ can be calculated as

(18) $$\begin{array}{l} W_{m}^{\left(0\right)}=\frac{\lambda }{n+\lambda }\\ W_{c}^{\left(0\right)}=W_{m}^{\left(0\right)}+(1-\alpha^{2}+\beta )\\ W_{m}^{\left(i\right)}=W_{c}^{\left(i\right)}=\frac{1}{2(n+\lambda )},i=1,\ldots ,2n \end{array}$$

where the parameter β is always chosen as β=2 for Gaussian distributions.

With unscented transformation, more training data can be obtained based on the primary data. However, the definite form of this nonlinear transformation is undetermined, we must assume one. Considering that the predicted secondary data need to be IID with the CUT, a linear transformation y=kx+b which will keep the homogeneity of the data are used here. This linear transformation could be seen as a generalized nonlinear transformation.

### 4.2. Algorithm Procedure

The procedure of the proposed single-dataset-based preprocessing JDL algorithm is described as follows:

1. Determine the size of the LPR and transform the receiving data to the angle-Doppler domain using Equations (6) and (7).
2. For transformed $\eta_{a}\eta_{d}\times 1$ data ${\tilde{x}}_{l},l=1,2,\ldots ,L$, conduct the unscented transformation to get $\left(2\eta_{a}\eta_{d}+1\right)$ sigma vectors $\left\{\sigma_{{\tilde{x}}_{l}}^{\left(i\right)}\right\}$ by using Equations (16)–(18).
3. Set the transformation y=h(x) as y=x for convince. Here we get $\left(2\eta_{a}\eta_{d}+1\right)$ secondary data $\left\{\sigma_{{\tilde{y}}_{l}}^{\left(i\right)}\right\}$. Calculate the weighted sample mean and sample covariance of $\sigma_{{\tilde{y}}_{l}}^{\left(i\right)}$ by using Equations (14) and (15). The result ${\hat{R}}_{y_{l}}$ can be seen as the initial estimate of the CCM of ${\tilde{x}}_{l}$.
4. Estimate the CCM ${\hat{\tilde{R}}}_{l}$ with the equation ${\hat{\tilde{R}}}_{l}=\frac{1}{L}\sum_{p=0}^{L}{\hat{P}}_{y_{p}}$.
5. Calculate the adaptive weights as Equation (11) denotes and calculate the output statistic by using Equation (12).

The second step and third step mitigate the influence of the nonhomogeneity and are based on the single data-set. This preprocessing approximates the CCM of the CUT through unscented transformation. Then the forth step integrates the effect of different range bins as a way of smoothing to increase the rank of the CCM, which will further decrease the effect of nonhomogeneity.

## 5. Experimental Results and Performance Comparison

In this section, the experimental results are shown using measured data to evaluate the proposed approach. The performance comparison with other existing clutter-suppression method for shipborne HFSWR proposed before is also given. The Results have verified that the proposed method outperforms other tested methods.

### 5.1. Measured Data with Simulated Target

To validate the performance of the proposed method for multitarget situations, three simulated targets are injected to the real clutter data, the detailed information is shown in Table 2. The signal-to-clutter-plus-noise ratio (SCNR) refers to the total clutter power in the target range cell after Doppler processing and before beamforming. It is worth mentioning that the Doppler frequency of the first target is close to the theoretical Doppler frequency of the first-order sea clutter when the azimuth is set to $10^{\circ }$. According to Equation (13), the Doppler frequency of the first-order sea clutter is −0.2039 Hz.

**Figure 7:**
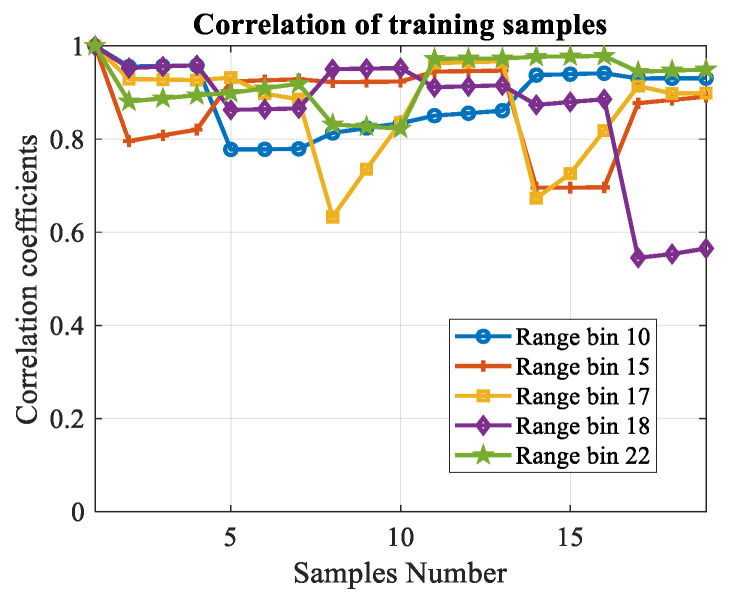
Correlation coefficients of the training samples.

As mentioned above, the unscented transformation is conducted on the primary data first. To prove the validity of the generated secondary samples, the correlation coefficients are calculated using the azimuth–Doppler joint eigenvectors analysis method listed in Section 3.3. Some of the range bins are chosen randomly to show the result. In our experiment, 32 range bins are used totally, and the local processing region is $3\times 3\;(\eta_{a}=3,\eta_{d}=3)$. For each range bin, the unscented transformation is conducted and $(2\eta_{a}\eta_{d}+1)=19$ training samples can be obtained. Figure 7 illustrates the correlation coefficients of the training samples produced from the primary data. Each line represents for one range bin. It can be seen that almost all of the coefficients are above 0.6, which means the produced training samples are homogeneous.

Some of the correlation coefficients are much lower than others. The reason is that the clutter to noise ratio (CNR) decreases in these selected LPR. In other words, the proportion of the first-order sea clutter is lower than other range bins. The proposed data acquisition method can be considered as a predictive modeling. The data in a certain LPR consists of three parts, target, clutter and noise. For the training samples, it is assumed that there are no targets. The correlation coefficients are determined mainly by the clutter since that the correlation of noise is much weaker than the first-order sea clutter. Hence, if the CNR decreases, the correlation coefficients will be unstable and some of them may be smaller.

**Figure 8:**
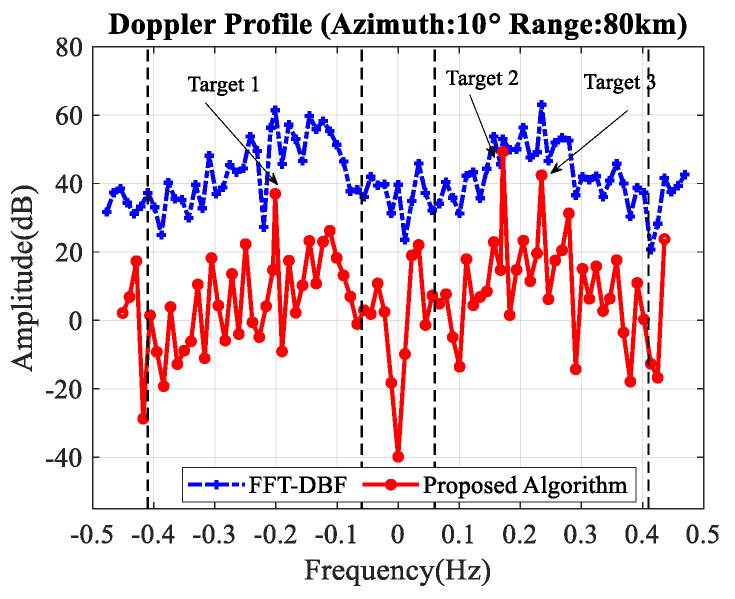
Doppler profile before and after clutter-suppression.

After the first step and second step, the third step and fourth step are performed. Figure 8 illustrates the Doppler profile when the range and beam are set to the target. The dash-dotted line marked with a plus sign indicates the initial Doppler profile without clutter-suppression. The solid line marked with a circle sign represents the Doppler profile after clutter-suppression using the proposed algorithm. The vertical dashed lines show the theoretical broadening area of the first-order sea clutter.

To evaluate the performance of the algorithm, the SCNR improvement, defined as the improvement of the SCNR before and after clutter-suppression, is given as follows:

(19) $$\mathrm{SCNR}=10\log_{10}(\frac{P_{s}}{P_{c+n}})$$

(20) $${\mathrm{SCNR}}_{\mathrm{Improve}}={\mathrm{SCNR}}_{\mathrm{after}}-{\mathrm{SCNR}}_{\mathrm{before}}\;\;\left(\mathrm{dB}\right)$$

where $P_{s}$ represents the power of the signal from the Doppler bin of interest, $P_{c+n}=\sum_{k=1}^{M}P_{k}$ represents the power of clutter plus noise, which is calculated as the mean of all the signal power from different Doppler bins in the corresponding clutter region expect for the bin of interest. For example, when calculating the SCNR improvement of target 1, the clutter-plus-noise level $P_{c+n}$ is measured from the negative first-order sea clutter part, while for target 2 and target 3, the positive broadening area is measured.

As shown in Table 3, all the three injected targets have a SCNR improvement after the proposed algorithm is applied. The average SCNR improvement is 19.2 dB. It is obviously that the target submerged in the clutter appears.

### 5.2. Measured Data with Real Target

A non-cooperative target is selected to investigate the effectiveness of the proposed method. It is a passenger liner named Changsong which was receding from the platform with the following parameters: range 78 km, azimuth $-5^{\circ }$ and radial velocity 5.68 m/s with the corresponding Doppler frequency −0.2 Hz. The sailing velocity and lane of the liner are quite stable [19].

**Figure 9:**
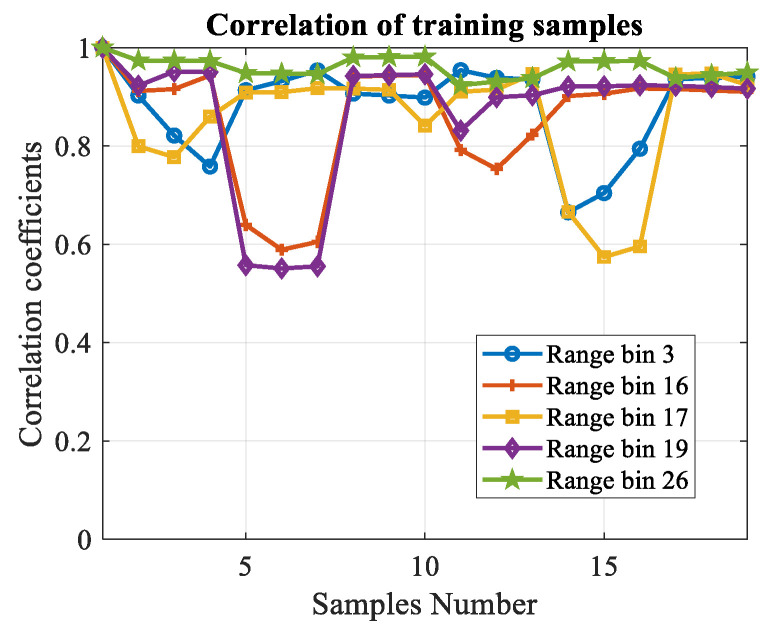
Correlation coefficients of the training samples.

The processing procedure is the same as simulated targets. Figure 9 shows the correlation coefficients of the obtained training samples. They are also almost all above 0.6.

**Figure 10:**
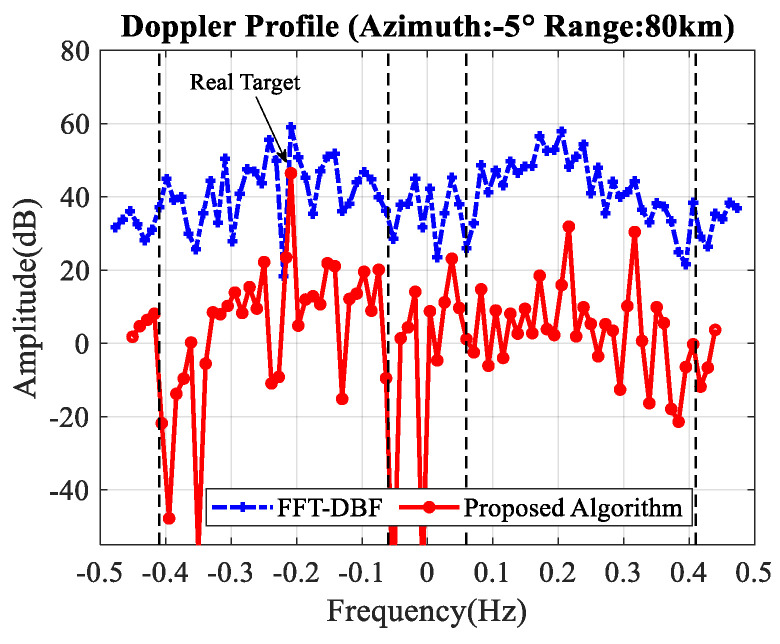
Doppler profile before and after clutter-suppression.

The Doppler profile is shown in Figure 10 when the range and beam are set to the target. The dash-dotted line represents the initial data without clutter-suppression while the solid line represents the final result. The vertical dashed lines show the theoretical broadening area of the first-order sea clutter. As illustrated in Table 3, the SCNR improvement reaches 24.9 dB and the target can be easily detected after clutter-suppression.

### 5.3. Performance Comparison with Different Algorithms

**Figure 11:**
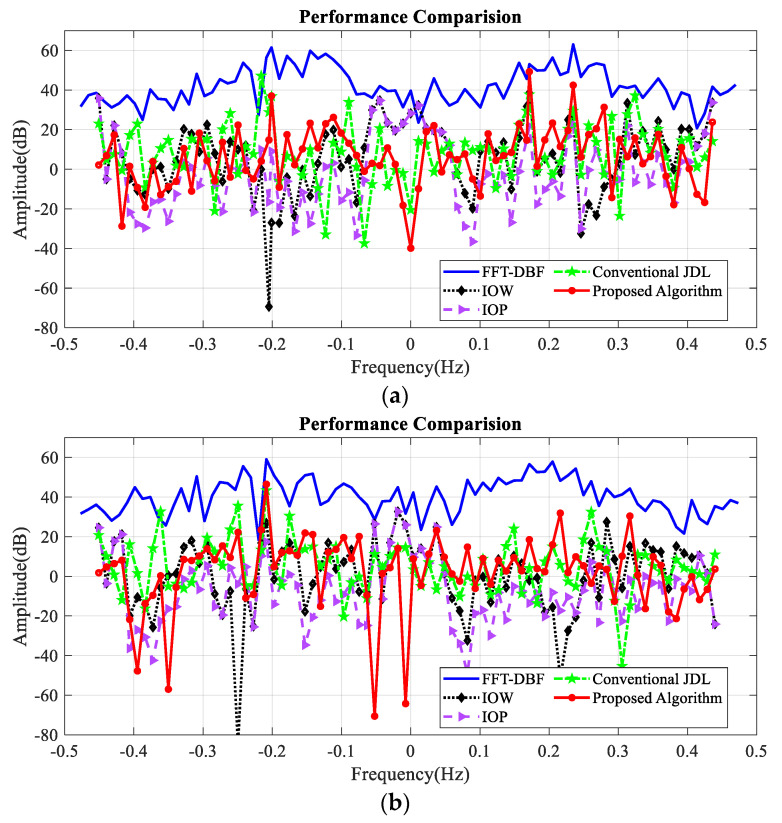
Comparison between different algorithm. (**a**) Simulated targets; (**b**) real target.

Figure 11 gives the comparison between the conventional clutter-suppression algorithms and the proposed algorithm (fast Fourier transform and digital beamforming (FFT–DBF), improved orthogonal weighting [26], improved oblique projecting [27], conventional JDL [24], and proposed algorithm) with three simulated targets and one real target, respectively.

Table 3 illustrates the SCNR improvement of these algorithms. As we can see, compared with other algorithms, the proposed algorithm has a higher SCNR improvement. For target 1 which is close to the first-order sea clutter, our proposed algorithm works well while the IOP algorithm and IOW algorithm fail. These two methods suppress the clutter through space nulling, when the targets are close to the clutter, there will be significant gain loss of the target.

## 6. Discussion

As depicted by simulation results given in Section 5, the proposed data acquisition method can generate 2 *N* + 1 training samples based on an *N* dimensional primary data. These generated training samples are highly correlated with the initial data that tested by the correlation coefficients, which means they are statistically similar. As shown in the result, the proposed algorithm performs well for clutter-suppression and increases the SCNR ratio, which will lead to better detection performance. Compared with other existing clutter-suppression methods like conventional JDL, improved orthogonal weighting, improved oblique projecting, the proposed algorithm has a greater effect on improving SCNR. The methods in previous work have an assumption that the spreading first-order sea clutter is statistically uniform in range dimension and ignores the heterogeneity of the clutter, our method analyses this property and provides a solution.

Furthermore, it is not only applicable for shipborne HFSWR, the proposed data acquisition method can also be used in all cases where the qualified training samples are limited. The proposed clutter-suppression method can be taken as an approach to solve nonhomogeneous clutter. However, the unscented transformation is used for nonlinear transform and here we use linear instead since the special nonlinear transform is unknown. If the statistics of the CUT is known, then how to find the most appropriate transformation needs further study.

## 7. Conclusions

In this paper, a single-data-based preprocessing JDL algorithm is proposed to suppress the spread first-order sea clutter in shipborne HFSWR. To deal with the impact of the nonhomogeneous characteristics and limited number of training samples, an innovative training sample acquisition method is proposed using the unscented transformation. Then, the CCM of the CUT is estimated by integrating different space–time snapshots, which will increase the rank of the CCM and mitigate the effect of the heterogeneity in range dimension. The real data are used to test the performance of the proposed algorithm. Experimental results show that the proposed algorithm has a higher SCNR improvement compared with FFT–DBF, improved orthogonal weighting, improved oblique projecting and conventional JDL algorithm and is applicable for first-order sea clutter-suppression in shipborne HFSWR.

## Figures and Tables

**Table 1:** System parameters.

Parameters	Symbol	Value
platform velocity	$v_{p}$	5 m/s
number of receiving channels	N	7
distance between sensors	d	14 m
carrier frequency	$f_{0}$	5.283 MHz
bandwidth	B	30 KHz
wavelength	λ	56.786 m
pulse repetition interval	T	0.262144 s

**Table 2:** Injected target parameters.

Parameters	Target 1	Target 2	Target 3
Range	80 km	80 km	80 km
Radial velocity	−5.71 m/s	4.87 m/s	6.66 m/s
Doppler frequency	−0.2012 Hz	0.1714 Hz	0.2347 Hz
Azimuth	$10^{{}^{\circ}}$	$10^{{}^{\circ}}$	$10^{{}^{\circ}}$
SCNR	0 dB	0 dB	0 dB

**Table 3:** Performance Comparison.

Algorithm	SCNR Improvement (dB)			
	Simulated Target 1	Simulated Target 2	Simulated Target 3	Real Target
Conventional JDL	9.0	19.1	0.75	17.9
IOW	−45.2	24.4	3.5	14.7
IOP	1.49	18.2	9.1	15.5
Proposed algorithm	14.3	30.0	13.2	24.9
